# Dental Caries, and Its Relations to the Germ Theory of Disease

**Published:** 1885-02

**Authors:** G. V. Black


THE
AMERICAN JOURNAL
OF
DENTAL SCIENCE.

VOL. XVIII. THIRD SERIES—FEBRUARY, 1885. No. 10.

ARTICLE I.

### DENTAL CARIES, AND ITS RELATIONS TO THE GERM THEORY OF DISEASE.

BY G. V. BLACK, M. D., D. D. S.

[Read in the Section on Oral and Dental Surgery of American Medical Association, May, 1884.

Caries of teeth has been defined as a molecular disintegration of the tooth substance; or a breaking down of the chemical constituents of the tooth, molecule by molecule. This destruction always has its beginning on the surface of the tooth, or in some pit, crevice or other imperfection in the enamel. And it spreads from this point, as the focus, in every direction, the dentine being destroyed more rapidly than the enamel; hence it usually happens that the cavity is larger within on the surface of the tooth. Caries does not seem to be a simple solution of the tooth's substance; sometimes we find nearly all of the material removed from the cavity, in other cases we find the dentine reduced to a pulpy or semi-glutinous mass, in which the structure of the dentine is more or less perfectly preserved. Some decays are white, some are black, some have a yellow tinge. All the shades from white to black may be found. But it was not my intention to give a lengthy description of the results of caries; but rather to confine myself as closely as possible to the discussion of the probable cause of the affection. I say probable cause, for I do not assume that the cause, or causes, are certainly known. Indeed, we may say that at the present time there is the greatest disagreement, among even the best informed men, on this important subject; and that it is still very uncertain that any of the theories in regard to the matter are correct. It is proper that I should say, however, that more than one of these explain the phenomena with sufficient accuracy to be of great value, both in the prevention and treatment of this affection. It must not be supposed that a theory must be absolutely correct to be of use. Theories are usually contrived in the effort to explain phenomena, and it often happens that a false theory leads to as good an application of means to ends as the true one would do. Of this, however, we can never have assurance; therefore, as long as there is a reasonable doubt, the search for the truth should continue.

The theory for the explanation of caries which has received the greatest attention, and the widest recognition in modern times, is what is known as the acid theory. This theory seems to account for the phenomena more perfectly than any other that has yet attained prominence in the minds of thinking men. As a working theory, a basis upon which to found principles of treatment, it has undoubtedly been the means of much good. Yet, in the scientific aspect of the subject, there is much objection to be urged against it. A very large amount of work has been done with the view of demonstrating the absolute truth of this theory; all of which must be regarded as a failure, so far as the attainment of that particular object is concerned. The labor has not been lost, however, but on the other hand has been of immense value. It is this labor, the basis of fact which it has brought to light, that will be of most service to us in the building up of other theories for the explanation of the phenomena, which may serve us usefully until such time as theories shall be displaced by demonstration; the goal to which we are all looking forward.

According to the chemical theory, the substance of the tooth is decomposed by an acid; this acid acts more readily on dentine than upon the enamel, therefore the tendency to the enlargement of the cavity toward the internal portions of the tooth. Some writers, as Dr. Watt, have attempted to define the acids thus acting, and to divide decays into classes according as this or that acid is active in its production. The acids that have been thus pointed out are, nitric acid (white decay,) sulphuric acid (black decay,) and chlorohydric acid (intermediate colors.)

Most of those who have written on this subject, however, have been content without specifying the particular acids so definitely; while some have been of the opinion that these particular acids have little or nothing to do with the matter, and seek to show that other acids are more likely to do the ugly work.

The views of Dr. Watt have received much commendation in this country, but not so in the old. There, other men have been prominent, and we find that while they have agreed in the main, there are important differences between them. As we have said, Dr. Watt has maintained that decay is caused by the acids—nitric, chlorohydric, and sulphuric, with possible others.

In Europe the influence of these particular acids has been very generally denied, and the results attributed to other acids, as the lactic, acetic and the group known as the organic acids. Among those that have examined this subject, none, perhaps, have attained a wider hearing than Magitot, of Paris. This gentleman published a work on this subject in 1868, in which he makes an extended examination of the subject, arriving at the conclusion that decay is caused by acids. These acids, however are derived from the saliva through the process of ferment action. Dr. Magitot instituted a long series of experiments to determine the effects of the suspected acids on the teeth. This series of experiments shows that most of the organic acids act very feebly on the teeth in the proportion of one to one thousand of water; and that in the proportion of one to one hundred they act quite energetically; so that the teeth submitted to their action will be completely decalcified within a few weeks or months. Most of this series of experiments were continued, however, for two years. The conclusion seems to be that caries may be produced by any of the group of acids that may be developed by the fermention of the saliva. These are the lactic, acetic, butyric, etc.

Dr. Magitot states distinctly that the agency of micro-organisms in the production of these acids is admitted by him; but discusses this phase of the subject no further. He makes no effort to determine to what extent these acids may be formed in the mouth. All of his experiments were tried out of the mouth, and no provision whatever was made to ascertain the effect that fermentation may have had on his solutions in the progress of his experimentation. This being the case, the only result of the experiments is the determination of the strength of the solutions of these different acids, necessary to decalcify a tooth.

This, together with the fact obtained from other sources, showing that most of these acids are the products of certain fermentations that may go on in the mouth, gives much force to Dr. Magitot's couclusion.

Very soon after Dr. Magitot's work, in the same year, indeed, came the work of Lieber and Rottenstein, to which we have referred. The work seems to have been written for the express purpose of showing that decay of the teeth is caused by the life and development of the fungus known as leptothrix buccalis. In this the authors seems to have signally failed. They certainly make but little advance toward the demonstration of the parasitic theory of this affection. Indeed they do not seem to have endeavored to show that this fungus does more than promote decay that has already been established.

They say, after having made an extensive examination of the life and growth of the leptothrix, "From what has been said it results that two principal phenomena manifest themselves in the formation of dental caries, viz., the action of acids, and the rapid development of a parasitic plant, the leptothrix buccalis."

They do not suppose the leptothrix buccalis capable in itself of attacking the teeth, if their condition be normal, but when their surfaces are once softened by acids, then the fungus may penetrate the portions thus softened, and continue the destruction.

Again they say: "It seems that the fungi are not able to penetrate an enamel of normal consistency. The dentine itself, in its normal condition of density, offers great difficulties to their entrance, and we are not yet sure that the leptothrix could triumph over this resistance," Again: "We cannot decide at present if the leptothrix is able to pentrate sound dentine when, from any circumstances, it happens to be denuded." * * * "But if the enamel or dentine are become less resistant at any point, through the action of acids; or if, at the surface of the dentine, a loss of substance has occured, then the elements of the fungus can pass into the interior of the dental tissues, and produce by their extension, especially in the dentine, effects of softening and destruction much more rapid than the action of the acids alone is able to accomplish." * * * "The participation of the fungus is constant in the progress of caries which have reached this stage. As soon as a loss of substance can be shown there is found the presence of the fungus, so that the question whether or not the acids alone could produce ravages more considerable is without importance."

The modus operandi by which leptothrix may produce softening of dentine is left without explanation. We can conceive, however, that they may do something to assist the softening process by the out-pouring of a digestive fluid. If, however, this fungus gave a fluid that would digest a tooth, we would think that sound teeth would be very scarce, for its grows abundantly in every mouth.

Since the time of Lieber and Rottenstein's work we remember of no other of much importance having appeared on this subject. The discussion has continued, however, in the journals. We cannot undertake to review this literature, interesting as it would be; but we must content ourselves with one writer, Dr. Miller, now of Berlin.

Dr. Miller's experiments bear the stamp of being more carefully performed than any that have previously come to our knowledge. This was to have been expected from the fact that they are the latest, giving the experimenter the advantage of all that has gone before, and for the reason that he is very favorably placed for such work, being in the midst of the best experimenters of the world. Therefore his work is looked to with unusual interest.

We need not, however, notice any but his last series of articles, that which is now appearing. We cannot, of course, criticise Dr. M.'s work now, for we have not heard him through; but enough has appeared to show very clearly what the result will be.

Dr. M. begins this series of articles with this sentence: "During the last two years I have stated at different times and places, as the result of many experiments, that "the first stages of dental caries consists in a decalcification of the tissues of the teeth by acids, which are for the greater part generated in the mouth by fermentation. The object of the investigations described in this and the following papers is to determine this ferment, and the conditions essential to its action.'"

We see from this that Dr. Miller begins just where Dr. Magitot left off, sixteen years ago. The discussion of the subject during these years has given us no additional facts as to the essential nature of these phenomena; but the advance of thought in reference to the general subject of such investigations has been such that no man would now repeat M. Magitot's course of experimentation with the same end in view.

I will give you a very brie synopsis of Dr. M.'s course. And while I do so I wish you to keep in mind the object he has in view. It is admitted that decay is brought about by acids, developed, or Dr. M., supposes them to be developed, by fermentation of some kind. The object of this course of experiments is to find and examine this supposed ferment.

Is is not necessary that I should describe in detail all the apparatus with which the experimenter provided himself; it is sufficient to say that all the appliances for the prevention of error were used. The first question to determine was whether or not the ptyaline of the saliva could so change starch as to produce an acid. This question was soon decided in the negative. The starch was promptly changed into sugar, but here the rea-ction ceased; the fluid remained permanently sweet when the proper precautions were observed to prevent the ingress of germs. This proves that the acidifying power does not belong to the saliva. It must, then, be something foreign.

Now, a freshly extracted carious tooth was taken, all food removed, the outer portions of the decayed mass saturated with a 90 per cent, solution of carbolic acid to destroy any accidental germs that might be in this portion. Then, with an instrument, purified by heat after each cut, layer after layer of the softened dentine was removed until the inner portions were reached. Then a slice was quickly conveyed to a sterilized culture medium, composed of sterilized saliva, water, sugar and starch, and placed in an incubator, together with another test tube of the same culture fluid uninfected, to serve as a check. In twenty-four hours the infected culture became acid, while the other did not. This remained constant in a sufficient number of experiments to establish the fact that the acidity was due to the infection.

From the cultures that had become acid, other cultures were infected, which also became acid ; thus proving that the experimenter was dealing with a ferment that was capable of propagating itself; an organized ferment.

Microscopic examination showed that these cultures contained an organism similar to those found in the deeper layers of carious dentine, and which remained constant in their characters. Chemical examinations, which seem to have been carefully conducted, showed the acid produced to be lactic acid. This acid has been shown to be capable of decomposing the teeth by M. Magitot, and many others. Yet Dr. Miller goes still further, and by placing sections of dentine in his culture fluids that he has infected, finds that they are decomposed by the acid formed ; while such sections placed in such fluids not infected are not changed. Thus he not only proves that an acid is formed, but that the acid is formed in sufficient amount to destroy the dentine.

This, when compared with the best experimentation previously had, marks a great advance. One point seems to have been gained. One organism has been traced thus far, and may now be said to have proven to be able to produce certain of the phenomena of decay. But this is not all. There is much yet to be done. True, one other point is spoken of by Dr. Miller. All who have made a careful study of caries know that there is a peculiar enlargement of the tubules, which is not seen in dentines softened by acids alone. Dr. Miller has been looking for this also, and not without success, for in some sections of dentine exposed to the action of the cultures, he found the organisms crowding into the tubules, and tells us that he also found them enlarged as in natural caries of the teeth in the mouth ; indeed that he had before him veritable caries artificially produced.

This delineation of results fo experimentation must have great weight in the settling of the problems at issue, especially if they are confirmed by other competent observers. There is nothing in these experiments that are not in harmony with known facts, unless it be the widening of the tubules by the crowding in of the organisms. It is known by previous experiment that this widening is not caused by the lactic acid as it exists dissolved in the surrounding medium, and I think very few will be willing to concede that the organisms can accomplish this by physical force. This point requires further discoveries. However, I think it may be explained in advance; at least the effort may serve to direct experimentation.

In a previous lecture I have dwelt at some length upon the digestive fluids of living organisms. Unexpectedly I find use for these ideas now, for they were written before I saw Dr. M's last article. I have explained how it is that dead bone, roots of the temporary teeth, ivory driven into the flesh, catgut ligatures, sponge, etc., are dissolved and removed by a soluble ferment. I have also shown that the soluble ferment of the yeast plant has been found and proven. Also that of ammoniacal fermentation; and how plants take up otherwise insoluble substances. Now, this widening of the tubules is a conceded fact. It is also shown that it is not done by the lactic acid in case of other experiments, nor can it be done by the lactic acid in case of other experiments, nor can it be done by the physical force of the organisms. but it can, in all probability, be done by the digestive fluid of the organism. The conditions for this work of the digestive fluid are the same as that of the granulations in widening the meshes of the sponge and finally removing the last of it. As yet no soluble ferment has been demonstrated in connection with this organism; but theoretically it must exist, and if Dr. Miller should undertake to search for it, he will be able to demonstrate it speedily, and its co-operation in producing some of the phenomena of decay; at least determine its capabilities. This organism cannot be said to have been definitely and completely studied until this soluble ferment, or diastase, be found, isolated, and its capabilities separately determined. (Since the above was written Dr. Miller has reported the finding of this soluble ferment.)

It is by no means probable that this is the only organism that may stand in a causative relation to caries.

(Since the above was written, Dr. Miller has also reported the finding of another micro-organism that he thinks capable of producing caries.)

The organism of butyric fermentation, possibly that of acetic fermentation, and a large number of others of the acid fermentations, may cause decay; nor is it by any means a settled fact that decay of the teeth may not be brought about by other vital processes than the acid fermentations. Of this, however, we will speak later.

Another question may arise in this matter and need explanation. I have repeatedly said that the waste products of an organism prevented the activity of that organism when collected in a certain amount. How then can this organism continue to thrive in its own waste product, and thus continuously promote caries, by furnishing more, and still more, of this waste product? Simple enough. Every chemist who has studied lastic fermentation has been in the habit of introducing some form of lime into the fermenting fluid to "flix" the lactic acid in the form of a lactate of lime, in which case it does not hinder the progress of the fermentation. In this way a much larger amount of the lactic acid may be obtained, as it is readily regained from its salts. This was learned long before this organism was found. Now, in the production of caries, the tooth presents the lime for the formation of the lactate, and thus furnishes the very conditions necessary to the continuous growth of the organism.

I wish now to turn your attention for a moment to another class of phenomena, and make some inquiry into their possible participation in the production of caries. I have already spoken of the strong probability that the otherwise normal tissues, when under the influence of certain qualities of inflammation, emit a fluid of acrid and very irritating properties. About the neck and other parts of children we sometimes see this fluid exoriating the skin wherever it touches it, seemingly acting the part of a caustic. I have already referred to the fact that dead bone, ivory driven into the flesh, sponge, catgut ligatures, etc., are dissolved and removed. This causes us to inquire whether or not a substance may be elaborated in the same manner in the mouth, that may have its quota of effect in the production of the phenomena of caries.

I was thoroughly convinced that this was the case years ago, though I endeavored to explain the results by the assumption that an acid is formed. This is entirely unnecessary. Soluble ferments do not seem to depend for their action on either acidity or alkalinity. They seem to be controlled by some other than the known chemical laws, and their action is not yet understood. We have no means of explaining them. If a piece of ivory thrust into the flesh is attacked and burrowed out in holes by a secretion thrown out by virtue of the irritation induced, as asserted by Krause, Kolliker, and other of the most capable observers of the world, why may not a tooth be attacked in the same way by virtue of an irritation of the tissues about its neck, or during the irritation consequent upon its eruption when this is usually prolonged? As a matter of fact, it has been observed that decays are prone to occur in just such situations as tend to confirm this hypothesis.

Thirteen years ago we drew attention to this, in a paper before the Illinois State Dental Society. Both before and since that time I have given much attention to this point, and I am more than ever convinced that it has much to do with the beginning of decay. I do not wish to be misunderstood in this matter. It is not my notion that decays are initiated by this cause alone. It is only one of the first steps by which other forces which come later are rendered operative—a means, if you please by which the surface of the tooth is first broken, and by which organisms are permitted to find lodgment; not means by which decay is carried on, to the complete destruction of the tooth. This effect cannot be produced except while the tissues are in contcat, or in very close proximity to the part in the process of solution, for the reason that the secretion from the tissues would be dissipated in the fluids of the mouth before they could have time to produce their effects upon the tooth structure.

The disposition at which these results are seen are: Wisdom teeth that come through very slowly, on the buccal surfaces of the molars generally, and sometimes on the labial surfaces of the upper incisors. In case of the wisdom teeth, the fact that they are very often decayed before they are fully through the gum is especially remarked; and as a rule, if these decays are carefully noted at a very early period of their progress, it will be seen that they are different from other decays in several respects. It always has its beginning under the free margin of the gum. There is usually no change whatever in the appearance of the tooth; the eye discovers nothing. The surface seems normal, or at most the portion of the tooth appears rather whitish; but on trial with the excavator the instrument will apparently break in through the enamel prisms, disclosing a cavity of very slight depth. It often happens that the enamel may be easily scraped away over a considerable space, as though it was so much chalk. The depth will present much variation; often it is only a part of the thickness of the enamel; at other times we may find it extendng into the dentine, in which it forms a veritable cavity If there is much depth, however, the characteristics will have assumed the more usual type.

Occasionally we see this character of decay (if it may be so called) in the grinding surfaces of the wisdom teeth; occasionally in the first molar, also, where the tooth has come through very slowly, and the gum has been for a long time in a state of chronic irritation.

It is characteristic of this effect that it is often seen on the smooth surfaces of the teeth as in the pits and grooves. No imperfection is necessary to prepare the way for this manifestation.

As I have said this takes place under the gum; is covered by the gum Now, as the tooth rises higher and the surface thus affected becomes exposed, these spots are prone to become the seat of true caries with all of its usual manifestations. However, very often caries does not take place. In this case, a whitish spot is seen, which gradually assumes a yellowish tinge, then brownish, and then finally becomes black. This result is brought about by the settling into, or the formation in, the affected tissue of the black sulphurets; as I was the first to show. (See report on dental chemistry, by Dr. H. A. Smith, Transactions of the American Dental Association, 1874, page 78.)

We see these spots every day upon the molars in every stage of coloration, from ashy white to a deep black. They are as apt to be on the otherwise smooth surfaces, as in the pits and grooves. They may occur on any of the teeth, but are oftenest seen in the positions named. The decays that so often occur on the labial surfaces of the upper incisors are, often though not uniformly, of the same character.

Those decays that occur just at the junction of the enamel and cementum, in persons of middle age or past, occasionally in younger persons, seem, in very many cases, to be of the same character. Some irritation of the gum at the immediate spot seems to be one condition of their beginning. These also have some special characteristics not common to other decays. If they are closely examined very easily in their inception, it will be found that the cementum has been removed, and that the margin of the enamel has become chalky; soon after this, if the case continue to progress, the gum, which till now was closely applied to the part, becomes everted so as to expose the breach in the tooth. This often becomes the seat of the most exquisite sensitiveness just at the present stage of the process, which calls the attention of the patient to the spot. Generally, however, nothing can be seen by either patient or operator, except a slight eversion of the gum, and the slight grooved appearance of the neck of the tooth, which the operator is often puzzled to differentiate from the normal form of the tooth. However, if he will carefully press the gum away (and he will find this abnormally sensitive) until he can see the root of the tooth below plainly, it will be easy to demonstrate that there has been a decided loss of substance. Trial with an instrument will develop the fact that the surface within this groove is exceedingly sensitive; the dentine is exposed. Now if the case is left to itself, this sensitiveness will continue some weeks, or even months, and then abate: and it will be found that the case has taken on the usual charatcer of decay. It may cease to progress and assume a dark color, or it may progress rapidly, and remain of a more or less ashy cast.

It has been our opinion that this class of decays, if that term can be applied at this stage, is brought about in precisely the same way that the root of a permanent tooth is partially absorbed on account of a chronic irritation of its peridental membrane. In other words, a soluble ferment has been called out by the irritation, that has dissolved out a part of the tissue at that point. Or, if you prefer to have it put in that way, a true absorption has taken place which forms the nidus for the future decay.

Another class of decays are very common which I have studied very closely, and which seem to be of the same character in their inception. These begin under the free margin of the gum under plates that abut closely against the teeth. These are usually very rapid in their course, evidently for the reason that as soon as the free margin of the gum is everted, a pocket is formed by aid of the plate, in which fermentation can proceed to the very best advantage. It does not seem that the beginning of this decay is often after the eversion of the gum has uncovered the spot. Of course, we often see decays occur where clasps encircle the teeth high up on the crown. Such must not be confounded with those that begin at the margin of the cementum.

DISCUSSION ON DR. BLACK'S PAPER.

*Dr. Marshall.*—In Dr. Miller's experiments on dental caries, whether produced in the mouth or artificially in his culture fluids, he invariably found an enlargement of the dental tubuli, to the extent of the penetration of the affection, and that the tubuli were filled with micro-organisms.

Dr. Boedcker, of New York, found the same enlargement of the tuouli, cut minus the micro-organisms, in his experiments with regard to the action of arsenious acid upon healthy dental tissue.

Dr. Black refers the cause of the enlargement in Dr. Miller's experiments under both conditions, to the action of a soluble ferment produced as a waste product by the *bacterium lactis*, and which dissolves or digests the organic portion of the tubuli, and the inorganic material surrounding it, while Dr. Boedcker refers the condition found in his experiments to inflammatory action resulting in absorption caused by tne irritation of the arsenious acid.

I would like to ask Dr. Black whether he considers the conditions found by these gentlemen to be identical?

*Dr. Black.*—I will first say that I do not regard the results as caused in both instances by the *bacteriumu lactis*, but in both cases the results are brought about, in all probability, by a digestive body. The action in both cases is the same kind, the philosophy of the widening is the same in both instances, the different forms of life. In the case of Dr. Boedecker's experimeuts, the conditions described could only be due to the formation by the irritated dentinal fibrils of a menstruum which digests and removes the tubular wall.

In Dr. Miller's experiments, the bacteria form a menstruum which digests and removes the walls of the tubuli.

In the case of the *bacterium lactis*, a digestive body, or diastase, has now been demonstrated by Dr. Miller, the organism is fully proven to be able to perform the act of digestion, and we know that the tissues of the higher animals have the power of removing parts by what is usually called absorption, and which is a form of digestion.

It has been well shown that the pulp chamber may be enlarged in this way in cases of long-continued irritation of the pulp. Bone is also easily renoven under pressure, evidently by a similar vital process—a process of digestion by means of a digestive body.

*Dr. Allport.*—Dr. Boedecker's experiments with arsenic upon dentinal tissue (though not yet fully completed) were upon living tissue, and evidently prove that there was inflammatory set up in the tubules, causing absorption of material. Dr. Miiler's experiments were upon dead tissue (teeth out of the mouth,) and the action seems to have been a chemical one. In the first, the process was a vital one, and may be in kind like that manifested in the absorption of the roots of deciduous teeth, the removed material being largely taken into the general circulation, which may have been "re-molecularized" and converted into new tissue, or carried off as a waste product. While, in the other case, the action was entirely from without, and would seem to have been from acids, and independent of visal processes within the tissues. I can imagine how micro-orginisms may produce a sufficiently exalted irritation of the dentinal fibres to cause absorption of material similar to that produced by the irrition of arsenic in the experiments of Dr. Boedecker.

But in either of these conditions I should expect the changed material would be largely carried off in the general circulation, the same as it is in the enlargement of the pulp cavity consequent upon continued irritation of the pulp, as suggested by Dr. Black, or the absorption of the roots of the deciduous teeth; in both cases the organic and inorganic materials are removed entire, while in caries occuring in the mouth the inorganic material only is largely removed, the organic portion remaining behind to die, be decomposed and washed away, or pass off in gases.

In the experiments of Dr. Miller the conditions are entirely external, and a purely chemical action, resulting in a single change of the elements, and their removal consequent upon the affinity of lime for acids. Whether the tungi excrete an acid or not (and more than likely they do) is of no material consequence so long as the acid is found there; it is the affinity of lime for acids, no matter how produced, whether from a decomposition of foreigh matter, or excreted by the fungi, that to my mind, produces the changed condition in the devitalized dentine

I have, of course, been interested in the arguments of Drs. Black and Miller. They are highly interesting, and there is little doubt that we are approaching a correct solution of this vexed question ; but it will be well for us to pause before we pronounce too confidently that either of these gentlemen account for all the phenomena of dental caries.

*Dr. Williams.*— May not the enlargement of tubules be caused by the physical force of the micro organisms exerted upon the walls of the tubules? In the vegetable kingdom such instances are not of rare occurence; rocks have been split, and large masses of solid material moved by the continued pressure of fungous growths.

*Dr. Fridericks.*—Milles and Underwood first called attention to the enlargement of the dentinal tnbuli, and they presented microscopical specimens to substantiate their views. Dr. Boedecker's experiments do not prove anything. I wonld not put much reliance upon the observations of two experimenters. I think we are begging the question.

Some observers can see whatever they desire to see, and later their observations are proved to be worthless. May not the enlarged appearance of the tubuli be due to the methods used in preparing the slides?

*Dr. Allport.*—I would snggest that Dr. I. L. Susserott, of Chambersburg, Pa.. be invited to speak upon this subject.

*Dr. Susserott.*—I am very much pleased with the paper just read by Dr. Black, it proves the truth of the lines by Butler, that

"Large fleas have little fleas,
And these have fleas to bite 'em;
And there are fleas to feed on these,
And so *ad infinitum.*"

Various remedies have been introduced for the antiseptic treatment of surgical diseases, the most potent of which is bichloride of mercury. As used in general surgery, a solution of 1 to 1,000 is perfectly harmless, and I do not see why it might not be used with safety and snccess in the treatment of dental caries.

*Dr. Harlan.*—The bacillus anthrax and the bacillus tuberculosis can live in absolute alcohol, and also in a five per cent. solution of chloride of zinc.

In order to know how to destroy micro-organisms, we must study the individual organism and apply the special agent which will destroy its vitality.

Eugenol—in the strength of 1 in 75,000, will destroy bacteria, and a weak solution will prevent the development of the spores.

*Dr. Black.*—I quoted an expression of Dr. Miller's saying it was unfortunate, viz.: "The first stages of dental caries consists in a decalcification of the tissnes of the teeth by acids, which are for the greater part generated in the mouth by fermentation." This is the same question discussed of old, of who led the pig to market, the man or the string. The string is the means used by the man, and the acid can no more cause decay, or even exist in that position, without the organisms, than the string can exist and lead the pig without the man.

The micro-organisms that produce caries must have free oxygen. Therefore, if you plug a tooth perfectly over a little softened dentine you exclude the free oxygon and destroy the organisms. Is is possible, of course, that an organism not requiring free oxygen might produce caries, but all the evidence we have against that supposition.

*Dr. Friedrichs.*—It seems to me that we are still begging the question. What is the need of antiseptic treatment of caries if micro-organisms cannot exist without free oxygen? When a cavity of decay is hermetically sealed no oxygen can enter, and consequently micro-organisms cannot develop.

*Dr. Marshall.*—It has been proved by Pasteur that there are certain forms of micro-organisms that exist without free oxygen, in fact cannot develop in that medium; and it is not at all improbable that such micro-organisms may exist as factors in the cause of secondary caries.

Dr. Black closed the discussion by saying: We must come to the point of finding specific micro-organisms. A poison for one form is not necessarily a poison for another. Only about 17 per cent. of alcohol can be produced by the yeast plant, because the excretory product of the plant—alcohol—in that degree of concentration becomes a poison to the plant and prevents further growth. This same alcohol, however is the natural food of the mycoderma aceti. This plant produces acetic acld, which in turn will stop the growth of its plant at a certain degree of concentration. This will now become the food of other organisms. So it is, that that which is poisonous to the one is not necessarily poisonous to another. Certain diseases occur in the child that do not occur in the adult. We believe some of these diseases are produced by micro-organisms. There is some difference in the nature of the tissues or fluids in the two cases that renders the adult unfavorable soil for the growth of these organisms. This gives some hope that in the continuous round of research some means may be found of antagonizing micro-organisms without a resort to poisons, as is now done. These will certainly be different for different organisms.

On motion it was agreed that owing to the lateness of the hour the reading of the paper by Dr. Harlan be deferred, and that it be the first order of business at the session to-morrow.

The section then adjourned.—*Jour. of Am. Med. Asso.*

